# Environmental Signals on Microglial Function during Brain Development, Neuroplasticity, and Disease

**DOI:** 10.3390/ijms21062111

**Published:** 2020-03-19

**Authors:** Luana da Silva Chagas, Poliana Capucho Sandre, Natalia Cristina Aparecida Ribeiro e Ribeiro, Henrique Marcondes, Priscilla Oliveira Silva, Wilson Savino, Claudio A. Serfaty

**Affiliations:** 1 Laboratory of Neural Plasticity Neurobiology Department, Biology Institute, Federal Fluminense University, Niteroi 24020-141, Brazil; luana_chagas@id.uff.br (L.d.S.C.); polianasandre@gmail.com (P.C.S.); nataliacristina@id.uff.br (N.C.A.R.eR.); marcondes.hm@gmail.com (H.M.); priscillaneuro@gmail.com (P.O.S.); 2 Laboratory on Thymus Research, Oswaldo Cruz Institute, Oswaldo Cruz Foundation, Rio de Janeiro 21040-360, Brazil; 3 National Institute of Science and Technology on Neuroimmunomodulation –INCT-NIM, Oswaldo Cruz Institute, Oswaldo Cruz Foundation, Rio de Janeiro 21040-360, Brazil

**Keywords:** neuroinflammation, brain development, immune cell cross-talk, neuroplasticity, environmental factors, microglial activation

## Abstract

Recent discoveries on the neurobiology of the immunocompetent cells of the central nervous system (CNS), microglia, have been recognized as a growing field of investigation on the interactions between the brain and the immune system. Several environmental contexts such as stress, lesions, infectious diseases, and nutritional and hormonal disorders can interfere with CNS homeostasis, directly impacting microglial physiology. Despite many encouraging discoveries in this field, there are still some controversies that raise issues to be discussed, especially regarding the relationship between the microglial phenotype assumed in distinct contexts and respective consequences in different neurobiological processes, such as disorders of brain development and neuroplasticity. Also, there is an increasing interest in discussing microglial–immune system cross-talk in health and in pathological conditions. In this review, we discuss recent literature concerning microglial function during development and homeostasis. In addition, we explore the contribution of microglia to synaptic disorders mediated by different neuroinflammatory outcomes during pre- and postnatal development, with long-term consequences impacting on the risk and vulnerability to the emergence of neurodevelopmental, neurodegenerative, and neuropsychiatric disorders.

## 1. Introduction

Microglia are mononuclear phagocytes known to play an important immunological and homeostatic role in the central nervous system (CNS) [1]. They are self-regenerative cells resident in the CNS parenchyma that differ from other non-parenchymal macrophages due to their origin [2]. While microglia originate from myeloid progenitors of the primitive yolk sac, macrophages derive from monocytes originating in peripheral embryonic blood vessels [3]. Despite this, studies with bone marrow radiation in chimeric mice have shown that, under pathological conditions, circulatory myeloid cells can be recruited, invade the brain, and assume a morphological phenotype that resembles the resident microglia [4].

During early brain development, microglial cells display an ameboid profile, with large and rounded cell bodies, short and thick branches, increased phagocytic activity, and specific secretion and gene expression signatures. At mature stages of development, microglia present a highly dynamic, reactive, and ramified morphology, immediately reacting to any alteration in homeostasis [5].

In addition to its role as nervous system sensors, being the first cells to respond in conditions of injury or infection, microglia have a well-established physiological role in the healthy brain, especially during earlier stages of development. During the prenatal stage, microglia are implicated in the induction of neonatal apoptosis [6], neurogenesis [7], promotion of neuronal fasciculation, and limits to axonal outgrowth [8]. Also, it regulates the laminar positioning of neurons and the complexity of the vascular network [9]. In the perinatal stages, microglia support neuronal survival [10] and promote phagocytosis induced by the death of neuronal precursors and surplus neurons [11].

One of the most well described physiological roles of microglia occur during the postnatal stage of brain development. Microglia are essential elements in the process of natural CNS plasticity, actively involved in the use-dependent rearrangement of synaptic connections that sculpt functional neural networks. Microglia promote the formation [12], maturation [13], and selective elimination (pruning) of immature synapses [14]. Recent work has shown, in the hippocampus, a partial elimination or trogocytosis of presynaptic buds and axons by microglia [12]. Therefore, microglial cells are now seen as components whose activation becomes a requisite for proper brain development [15].

During adulthood, microglia have a continuous motility and actively act in the surveillance of the cerebral microenvironment, alternating the extension and retraction of its branches throughout the extracellular parenchyma. In the mature brain, microglia monitor and modulate neuronal circuits [16] and the resulting neuronal activity [17]. In addition, they control neurogenesis, maintaining the pool of oligodendrocyte progenitor cells (OPC) and normal myelinogenesis [18]. Studies in the optic tectum of larval zebrafish demonstrate that the microglia control the excess of neural activity when interacting with highly active neurons [19]. Whole-cell patch clamp experiments have demonstrated that the activation of dendritic NMDA receptors in a single neuron is enough to trigger the growth of microglial extensions, establishing a direct link between neuronal activity and microglial dynamics. In relation to the mechanisms involved in this physical interaction, mediated by electrical activity between microglia and neurons, it has been established that this regulation is mediated by the GluN2A subunit of the NMDA receptor [20]. Besides that, microglia shift from different stages of activation also depends on a cross-talk between neurons, astrocytes, and microglial cells, which is essential for adaptive neuroplasticity. Neurons are able to inform microglia about their status, controlling their activation and motility through the secretion of soluble factors, extracellular vesicles, or contact-dependent mechanisms [21]. In the healthy brain, the surveillant microglia are under the control of neuronal factors, such as CD200 and fractalkine (CX3CL1) [22]. This interaction is also important for microglial colonization to the somatosensory, motor cortex, and hippocampus during early postnatal development, which is impaired in the absence of fractalkine signaling [23]. Immune-related soluble factors such as neurotrophins, neuropeptides, neurotransmitters, anti-inflammatory cytokines, and chemokines released by neurons affect microglia and promote specific microglia phenotypic changes [24].

In the last few years, a great discussion has emerged around the impact of sex differences on microglia phenotypes, function, and transcriptional properties. For example, a sexual dimorphic involvement of microglia has been shown in neuropathic pain signaling, where male mice sensitivity to pain is triggered by the activation of P2X4R on spinal microglia [25]; whereas in females, pain processing does not involve microglial cells [26]. Another study has also indicated sexual dimorphic response in an acute brain injury model that shows a faster activation and infiltration of pro-inflammatory myeloid cells to the male brain, but not in female mice [27]. A great number of neurological diseases present differential prevalence, incidence, and progression based on sex differences. Between the individuals that manifest neuropsychiatric or neurological diseases in any time of their lifespan, there is a susceptibility association, where females are related to diseases that occur during adulthood, and males are related to life-long neurodevelopmental derivative diseases [28].

Throughout life, environmental factors or stressful conditions, such as trauma or infections [29], hormonal imbalance [30], diet [31], and alcohol abuse [32], in early stages of development are related to synaptic disorders and increased risk for disease emergence. Importantly, all the above environmental impacts have already been demonstrated as modulators of the immune system, specifically modifying the microglial phenotype and function at the beginning of development. Herein, we present an overview of recent literature, supporting the impact of different environmental elements on the microglial phenotype and the consequences on appropriate brain development and neuroplasticity. We focus on nutritional, hormonal, and inflammatory imbalances, which are already known to impact microglia behavior, and the consequences on the shaping of neural circuitries, as well as the vulnerability to the appearance of neurodevelopmental, neurodegenerative, and neuropsychiatric diseases, such as autistic spectrum disorders and schizophrenia.

## 2. Neuroinflammatory Cross-Talk in Response to Brain Lesions and Plasticity

In the brain, the innate immune system is responsible for the detection and removal of invading microorganisms, senescent cells, surplus neurotransmitters, and aged and glycated proteins, which allows the maintenance of a healthy microenvironment [33]. Generally, in response to homeostatic disruption or signals released during normal development, these cells locally produce virtually all complement components, in addition to expressing complement receptors and the Toll-like receptors (TLRs) system [34]. Ultimately, microglia are able to orchestrate repair and homeostasis [35].

Pathological stimuli or cerebral trauma change microglial function, which stop patrolling the cerebral parenchyma to induce an adaptive inflammatory process. This response is characterized by a limitation of their motility, morphology, and function, transiently compromising the performance of essential physiological functions [36]. Such changes are associated with the release of chemokines, cytokines, and among them, some trophic factors [15]. In lesion conditions affecting brain lesions, microglial cells respond to damage migrating to the lesion site, where phagocytic activity removes cell debris and direct evidence for microglial activation in the fetus after an intrauterine infection pathogen [37].

Pathogen recognition receptors (PRRs) such as TLRs, present in macrophages and microglia, when activated by pathogen-associated molecular patterns (PAMPs) or tissue damage/cell death (DAMPs), induce the inflammatory phenotype [38]. These signals can be combined with inflammatory cytokines produced by Th1 cells, such as IFNγ. In vitro experiments have demonstrated that the stimulation of macrophages or microglia with LPS (+ IFNγ) triggers the classical activation pathway, or the pro-inflammatory profile [39]. Induction of the pro-inflammatory phenotype by LPS (+ IFN-γ) has also been demonstrated in primary human microglia [40].

The elimination of pathogens and cellular debris also occurs by microglial phagocytosis [41]. However, such responses may also affect neurogenesis and induce neurotoxicity through the release of oxidants, which in turn can activate an inflammasome [42]. In mice with persistent infection, microglia can act as a viral reservoir, presenting antigens that support CD8^+^ T-mediated cell viral elimination response [43]. In intrauterine infections, a direct evidence for microglial activation has been shown in a study with pregnant mice challenged with LPS: microglia presented an altered pattern of activation, with a persistent reduction of pro-inflammatory activation throughout the brain, whereas the hippocampal microglia presented an increased inflammatory response after a second challenge with LPS, negatively affecting learning and behavior in the offspring [44].

The relevance of T lymphocytes in brain plasticity has been shown in nude rats, transplanted with CD4^+^ T cells (but not CD8^+^ T cells), that were capable of presenting NT-3-induced axonal outgrowth in a model of spinal cord injury [45]. Therefore, a local inflammatory response results from production of microglial-derived cytokines and chemokines that recruit populations of peripheral immune cells, including T lymphocytes [46]. This is part of the adaptive immune response in cases of very severe brain injury, infections, or chronic diseases, when they require the participation of other immune cells [47,48].

Experiments mimicking immune infiltration into the healthy CNS in the absence of systemic inflammation are key to identify potential candidates that interact with T cells in CNS inflammation. Steady-state migration of conventional dendritic cells (cDCs) out of the CNS to cervical lymph nodes has been reported to be essential in immunosurveillance. Neuroinflammation associated with multiple sclerosis (MS) or with experimental autoimmune encephalomyelitis (EAE) is characterized by an increase in recruitment of cDCs to the CNS [49]. Using EAE as a model, Mundt et al. revealed that cDCs are critical for initial T cell reactivation and parenchymal infiltration [50]. In this case, cDCs could permit effective T cell–CNS interactions upon recognition and presentation of antigen for the T cells leading to their reactivation [51].

In turn, when infiltrating the brain tissue, T lymphocytes may influence microglial phenotype [52], determining different functions in early or late stages of aggression and healing. Indeed, a specific group of CD4^+^ T cells located in a niche at the choroid plexus acts as “gatekeepers,” mediating local and remote functions within the CNS territory, outside of the brain parenchyma, which is patrolled by microglia [51]. Those CNS-specific T cells have already been associated with the maintenance of functional neuroplasticity in the healthy brain [53]. These cells can facilitate the recruitment of other immune cells through the composite interface with the choroid plexus, by release of IFNγ [54], and to promote plasticity through IL-4 release [55]. Whereas the involvement of T lymphocytes occurs on demand of microglial activation, their activation could be one of the possible mechanisms by which microglia induce neural circuitry reorganization after an acute injury, and possibly in other pathological conditions of the CNS due to environmental changes, such as viral infections, malnutrition, endocrine dysfunctions, or toxicity by abuse of drugs.

### 2.1. Neuroinflammation and Brain Injury in the Adult CNS

Microglia and astrocytes are closely orchestrated as a team in brain lesions. The release of ATP by the damaged cells initiates microglial cell chemotaxis towards the lesion site by inducing an astrocytic ATP gradient, which is perceived by the purinergic receptor P2RY12. Microglia classically activated by a lesion or liposaccharide (LPS) induce astrocytic reactivity through IL-1β secretion, tumor necrosis factor (TNF), and C1q. Activated astrocytes not only facilitate activation of distant microglia, but also limit microglial activities [56]. While the lesion site is rapidly occupied by microglia, lesion borders are delimited by astrocytes that form a glial scar [57]. In addition, infiltration of macrophages and other immune cells also occurs at the lesion site [58]. According to the type of lesion and its extent, the fine regulation associated with the infiltration pathways and temporal window of action of these myeloid cells could promote its beneficial potential in the promotion of angiogenesis and axonal sprouting, in detriment of its deleterious role as the induction of a secondary degeneration [1].

Interleukin-1(IL-1), ATP, and transforming growth factor beta (TGF-β) are some of the molecules that mediate the interplay between microglia and astrocytes. For example, TGF-β, a fundamental regulator for microglia differentiation, promotes a unique transcription profile and surface structure of adult microglia [59]. The cross-talk between microglia and other cells of the CNS can also be mediated by CCL2, a chemokine mainly secreted by perivascular astrocytes, and important for microglia chemotaxis in many neuroinflammatory conditions [60,61]. Interestingly, a recent work from Xing, C., et al. proposes a gliovascular mechanism that regulates the microglial switch, where microglia assume different phenotypes in response to differential signaling from reactive endothelium that may, in turn, influence neuronal viability [62] factor (TNF) and C1q.

In traumatic brain injury (TBI) models, the altered permeability of the blood–brain barrier (BBB) results in increased infiltration of peripheral cells, promoting the exacerbation of tissue damage [63]. Several studies have demonstrated the presence of M1 and M2 markers (pro- and anti-inflammatory markers, respectively) in the “acute” phase, although in the subsequent “subacute” and “chronic” phases, the anti-inflammatory profile appears to reduce, while the pro-inflammatory profile remains, exacerbating the lesion [1]. However, studies in non-human primates have described a trophic, restorative microglial profile in the chronic phase of the lesion [64]. Indeed, many studies with different therapeutic approaches have demonstrated that the increase of anti-inflammatory markers is associated with neuroprotection, cognitive and histopathological improvement [65]. Interestingly, some studies have emphasized the presence of microglial activation for periods after injury. In a moderately controlled impact (CCI) model, it was observed a significant loss of cell branching and an increase in hypertrophic microglia in the mouse cortex one year after the injury, suggesting that although microglial activation occurs soon after the trauma, its phenotype and function may change over time and persist [66]. Persistent microglial activation after TBI has also been detected postmortem [67]. Other injury models, such as spinal cord injury, ischemic stroke, and macular degeneration, also have shown an imbalance between the pro- and anti-inflammatory phenotype, most of them favoring the M1, pro-inflammatory, profile [68].

In spinal cord lesion models, the lesion microenvironment favors macrophage/microglia polarization to the pro-inflammatory profile with a transient appearance of the anti-inflammatory profile shortly after the lesion. It has been observed that both pro- and anti-inflammatory markers upregulate shortly after injury, but three days after injury, pro-inflammatory markers continue to increase, while M2 profile markers downregulate, suggesting that the pro-inflammatory profile contributes to injury and inhibits axonal extension [69].

Although there is evidence that macrophages/microglia contribute to secondary tissue damage in CNS disease and injury, other studies report protective effects under these conditions. It has already been shown that microglia and astrocytes secrete BDNF, TGFβ, and FGF2 in traumatic spinal cord injury models, and such activity promotes neuronal survival, recruitment, and differentiation of oligodendrocyte precursor cells (OPCs) [70], indicating that an acute inflammatory response also contributes to pro-regenerative response with the activation of glial cells [71].

In the ischemia/reperfusion model, as well as in the spinal cord model, there is also the appearance of the microglial pro-inflammatory phenotype [72], which, together with neutrophils and macrophages, contribute to the neuroinflammatory cascade, propagating cell death beyond the initial ischemic region [73]. Despite the predominance of the M1-like response, the M2-like response also occurs simultaneously, regulating an exacerbated inflammation [74]. Rats that do not receive the appropriate signals for anti-inflammatory phenotype induction present worse outcomes after experimental cerebral ischemia, which is consistent with the idea that an anti-inflammatory response is required to repair and contain inflammation [68]. The deletion of galectin-3, a protein required for microglia activation, leading to a reduction in anti-inflammatory associated cytokines, such as IGF-1, results in a worsening of stroke-associated pathology [75].

### 2.2. Neuroinflammation and Microglial Function in Lesion Recovery during the Critical Period of Brain Development

**Figure 1:**
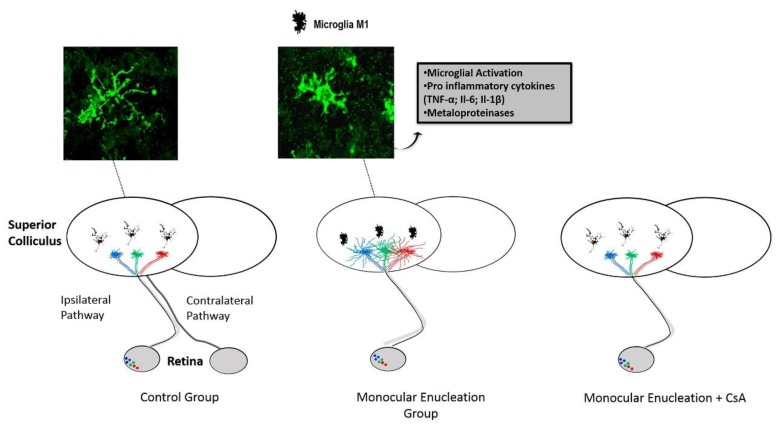
The rodent visual system reveals the effect of microglial activation on the modulation of the regenerative capacity of axons from the intact eye axons following a neonatal lesion (monocular enucleation) during early postnatal development. Under normal conditions, the retinal axons that form the ipsilateral pathway make connections to specific regions of the superior colliculus (SC). Following a monocular enucleation at P10, an extensive contralateral SC denervation occurs, followed by rapid compensatory growth of the axons from the intact eye. This plasticity depends on microglial activation, as it is abolished by immunosuppressive drugs (cyclosporin A or minocycline) administered intraperitoneally. Adapted from [78] with permission from S. Karger AG, Basel.

**Figure 2:**
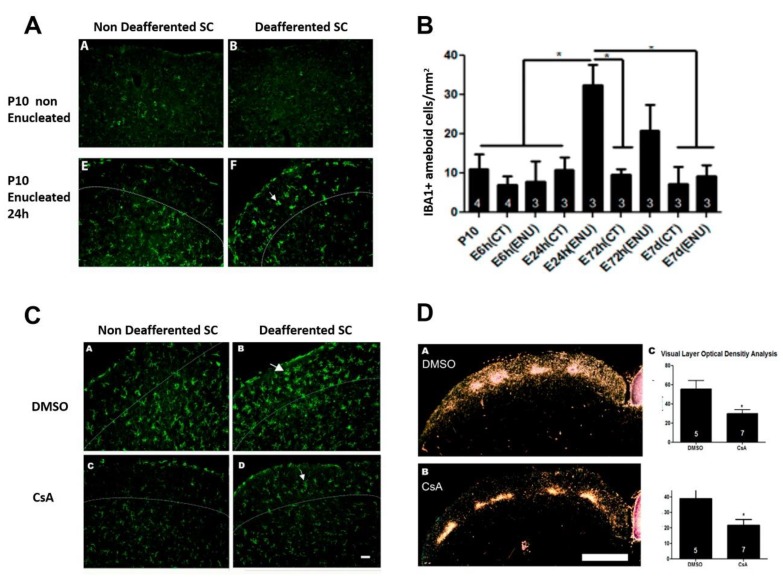
Inflammatory modulation following monocular enucleation in the rat visual system. Monocular enucleation induces a rapid phenotypic change in IBA1^+^ cells in the SC contralateral to the lesion, with the emergence of amoeboid cells 24 h after injury (**A**,**B**). Systemic treatment with Cyclosporin A (CsA) blocks the phenotypic change in the microglial population (**C**, lower right panel) compared to the control, vehicle-treated group (**C**, upper right panel). The SC not affected by the lesion can be seen in the left panels. Following monocular enucleation, immunosuppressive treatment also abolishes the plastic axonal growth of the uninjured eye axons (**D**). Figure adapted from [77], with permission from Elsevier, license no. 4724791219283 of 9 December 2019.

During brain development, plasticity occurs faster than that in adults [76]. For instance, in the visual system of pigmented rats, the removal of one eye during early development results in a rapid and long-lasting growth of axons originating from the intact eye, reaching a maximum level at 24 h after the lesion. This occurs simultaneously with a rapid microglial reactivity and migration to the visual layers of the colliculus (Figure 1 and Figure 2). Microglial activation begins with an increase in cell numbers, displaying an ameboid profile within 24 h (Figure 2), followed by a peak in the microglial colonization 3 days after the lesion. The reestablishment of the morphological profile, similar to the uninjured animals, occurred 7 days after the lesion [77]. In addition, the use of pharmacological blockers of microglial activation, cyclosporine A (CsA) (or minocycline), prevented microglial activation and axonal plasticity in this system (Figure 2). The same result was obtained after the local administration of a TNF-α neutralizing antibody, supporting that an inflammatory context soon after injury is a necessary condition for the promotion of adaptive plasticity and structural remodeling responses of the neural circuits, enabling a rapid recovery of the system in the early stages of development [77].

We also observed a post-injury increase of TNFR1 receptors (related to cell death induction mechanisms) and TNFR2 receptors (related to neuronal survival mechanisms) within 24 h, with return to control levels 1 week later (unpublished data), indicating that within a narrow temporal window after injury, inflammatory mechanisms occur in synergy, promoting both the elimination of injury factors, cellular debris, and tissue damage-related synaptic losses, but also acting in parallel, inducing repair and recovery of the system.

It has also been shown that plasticity induced by a monocular enucleation results, in same time-course, with an increased activity of metalloproteinase-9 (MMP-9) in the visual layers of the deafferented SC and the pharmacological blockade of MMP-9 blocks axonal plasticity from the non-lesioned eye [79], suggesting that a proteolytic activity is also necessary for triggering adaptive axon growth during development. Considering that MMP-9 can cleave not only extracellular matrix proteins, but also chemokines, it is conceivable that such a proteinase also acts on cell migration events.

Because of microglia’s dual role and diversity of functions and profiles over specific time windows that vary according to the specific injury type and the affected region, studies based on generalized microglial depletion are not considered effective therapeutic strategies. Accordingly, a more selective approach in the suppression of a specific microglial phenotype, in a proper time window and in a specific location, seems to be the current challenge of this field of research. Another point to be considered is the regenerative potential inherent to pro-inflammatory microglia, commonly associated with neurotoxic effects, since it has already been seen that the release of classic pro-inflammatory cytokines is directly associated with neuroplasticity and structural remodeling. The presence of both pro- and anti-inflammatory microglial phenotypes in the acute phases of a variety of different types of lesions raises the possibility of an important cross-talk between the pro- and anti-inflammatory profiles and the adaptive immune system. Further studies are necessary to address how a restorative outcome may occur in the absence of a secondary damage of the lesion environment with reduced risk for cognitive/functional decline.

### 2.3. Neuroinflammation and Microglial Function in Infectious Conditions

As mentioned above, microglia play a central role in synaptic remodeling and in the formation of neural circuits during early stages of brain maturation. Therefore, any failure in their proper physiological performance during these critical periods may result in the development of inappropriate neural networks, which result in the appearance of neurodevelopmental and psychiatric disorders [36] and in the pathogenesis of aging-related neurodegenerative diseases [80]. A recent study confirmed the existence of an age-related microglial phenotype during human brain senescence and its involvement in pathological processes associated with brain aging [81].

The microglial function on synaptic pruning and neural network formation prevails at the end of the gestational period and early postnatal development, whereas the acquisition of its “CNS macrophage” profile is only acquired at later stages [82]. Prenatal or perinatal infections appear to be a disruptor of microglial physiological functions, being an important environmental risk factor in the pathogenic processes of diseases such as schizophrenia [82] and autistic spectrum disorder (ASD) (reviewed in [83]). A mutant mice for CX3CR1, the fractalkine receptor, important in the maintenance of microglia in a non-activated form, exhibited a transient increase in the dendritic spine density of CA1 hippocampal neurons, associated with a temporary reduction in the number of microglial cells and accumulation of immature synapses, resulting in a lack of functional connectivity across the different brain regions with the presence of an autistic-like phenotype [84]. Also, the triggering receptor expressed on myeloid cells 2 (TREM2) seems to be essential for microglia-mediated synaptic pruning during brain development [85].

In addition, during pregnancy, fetal neurodevelopment is vulnerable to any environmental stimulus that could disrupt homeostasis, such as maternal infections. There are two barriers that protect the fetus from external pathogenic stimuli: The placenta and the BBB. In the late stage of pregnancy, the BBB is totally restrictive for maternal antibodies, for example, which could potentially cause damage to the developing fetal brain [86]. A recent animal study provided evidence that viral infections alone modulate the function of the developing BBB [87]. It is also known that various cytokines, as well as maternal leukocytes, cross the placental barrier [88]. The recent outbreak of Zika virus (ZIKV) infection in Brazil revealed a series of devastating consequences on fetal neurodevelopment that exemplifies how an environmental component like a virus can cause abnormal neurodevelopment, affecting the fetus’s immune system, possibly through changes in the maternal immune function, placental function, and microglia activity [89]. In the same vein, it has been postulated that during maternal infection, fetal microglia can be directly activated by some viruses, or indirectly through cytokines or microchimeric maternal cells [88]. Moreover, it has been shown that ZIKV invades microglial cells, promoting inflammation, thus disrupting their physiological role during brain development [90]. In addition to ZIKV, other viruses such as cytomegalovirus (CMV) and Rubella also cross the placental barrier and/or BBB and reach the CNS [89]. CMV infection of newborn mice induces a strong inflammatory response in the brain, characterized by microglial activation, recruitment of peripheral immune cells, and the expression of pro-inflammatory cytokines [91]. Thus, inflammation induced by viral infection is more responsible for neurodevelopmental abnormalities than the direct cytopathic effect of the virus on infected cells [92].

In an animal model that mimics a prenatal viral infection with the administration of policytidylic acid (poly I:C) in pregnant mice, in a period equivalent to the human third gestational trimester, changes were observed in hippocampal and medial prefrontal cortex architecture that contributed deficits in the cognitive and behavioral functions of the offspring. It was associated with an increase in the amount of hippocampal microglia, implying transient inflammation of the fetal or neonatal brain [93]. In another study, repeated systemic administration of the pro-inflammatory cytokine IL-1β in P1-P5 mice resulted in a transient increase in microglial density with long-term myelination deficits, followed by cognitive deficit [94].

Other models of pathogen infection have been associated with cognitive deficits and neurological symptoms caused by neuroinflammation mediated by microglial activation. In a recent work, it has been demonstrated that both neurotropic and non-neurotropic Influenza A viruses are able to promote long-term CNS deficits, suggesting that chronic CNS changes can also derive from infections. In this work, it was also demonstrated that the loss of hippocampal dendritic spines caused by the virus persists beyond the acute phase of infection and is directly associated with the increase in the number of activated microglia, reduction of the hippocampal long-term potentiation (LTP), and the resulting deficits in spatial memory formation, indicating a direct impact on synaptic plasticity [95].

The increased risk of ASD in children has also been associated with bacterial infections: Premature children with proven bacteremia in the first weeks of life have worse neurocognitive test scores [96] and present neurological dysfunction at school ages [97]. Also, parasitic infections such as *Toxoplasma gondii* have been associated with an increased risk in the development of ASD [98]. Together, these data strengthen the critical role of immune dysregulation during the critical period of development, predisposing to the onset of neurodevelopmental diseases.

### 2.4. Neuroinflammation in Fetal Alcohol Spectrum Disorder (FASD)

In addition to these mechanisms, other conditions that impact brain development can affect the microglial neuroinflammatory pattern. One such environmental condition is fetal alcohol spectrum disorder (FASD), which affects the children of women who drink alcohol during pregnancy and is currently the leading cause of mental retardation in the world [99]. FASD encompasses several pathologies and adverse effects caused in the fetus, ranging from neurocognitive and behavioral deficits such as learning deficits, reduced memory or visuospatial capacity, low behavioral self-control, rapid mood changes, attention deficit and impulsive behavior, loss of adaptive functions such as language and communication, poor social interaction, and difficulty in motor skills [100].

Studies indicate that ethanol exposure during pregnancy affects neural plasticity in the fetus. Specific cortical maps are altered in models of FASD [101]. Medina et al. showed in a model of monocular deprivation that ferrets exposed to alcohol present a decreased ocular dominance plasticity, preserving only more robust visual responses in the period between postnatal days 10-30 (PND 10–30), which is equivalent to the third trimester of human gestational period [102]. In addition to these findings, other studies have shown that alcohol exposure, even in moderate levels, reduces the dendritic tree formation and the density of dendritic spines in pyramidal neurons of the visual cortex [103] and the prelimbic regions of the frontal cortex [104].

Chronic alcohol exposure induces a significant increase of both pro- and anti-inflammatory microglial profiles in the hippocampus and the cortex of rats [105]. The pro-inflammatory signals induced by alcohol consumption appear to be mediated by TLR receptors expressed in microglial cells. It has been shown that the action of ethanol on TLR-4 can cause an increase of pro-inflammatory cytokines such as IL-1β [106]. Several studies, using in situ models and cell cultures, point to a positive correlation between a pro-inflammatory response during development and ethanol exposure [107]. Indeed, studies show that neuroinflammation plays an important role in pathologies associated with ethanol consumption. Drinking alcohol compulsively causes the increase of inflammatory cytokines in the circulation, both in healthy and unhealthy subjects [108]. A study observed that Iba-1 immunoreactivity increases in the cortex of animals that consumed ethanol for 12 months compared to animals that had exposure for only 6 months and animals that were not exposed, suggesting that the chronic consumption of ethanol induces a pro-inflammatory activation of microglia [109]. Also, it has been observed that the injection of ethanol in young rats (postnatal day 7) at concentrations of 3 and 5 g/kg, moderate and high doses, respectively, resulted in morphological changes typical of microglial activation 12–24 h after ethanol exposure [110]. Accordingly, Terasaki and Schwarz suggested that alcohol induces the expression of inflammatory genes in both the fetal brain and placenta [32]. Additionally, the ethanol exposure between PND 4–9 caused a reduction in the microglial population with a change to the ameboid profile, characteristic of an inflammatory profile, followed by the increase of the pro-inflammatory cytokines IL-1β and TNF-α, as well as the expression of CD11b [111].

### 2.5. Neuroinflammation in Congenital Hypothyroidism

Another congenital condition that is a main cause of non-genetic mental retardation is congenital hypothyroidism [112]. Thyroid hormones (TH) thyroxine (T4) and 3,5,3′ - triiodo- L- thyronine (T3) are essential for normal brain development [113]. Maternal TH deficiency around the 12th week of gestation is associated with a delay in the child’s cognitive and motor development [114]. Thus, congenital hypothyroidism, if untreated, may lead to developmental impairments such as motor deficits, mental retardation, deafness, and lethargy. These clinical manifestations reflect the involvement of TH in several processes of CNS development, such as neurogenesis, differentiation and neuronal migration, glial differentiation, synaptogenesis, and myelination [115]. Also, TH levels have an important role in structural and synaptic plasticity. For example, in the cerebellum, it has been shown a severe shrinkage of the Purkinje cell dendritic arbor in hypothyroidism [116].

The role of TH on the development and function of microglia has been uncovered over the last few years. Lima et al. demonstrated how hypothyroidism and hyperthyroidism may influence the development of microglial cells. The deficiency of TH from embryonic day 16 and during lactation resulted in a drastic reduction of microglial branches in cortical and subcortical regions of the rat brain. The morphological differences between the microglia of rats submitted to hypothyroidism and euthyroid rats were observed from the fourth postnatal day and remained until lactation (end of the third postnatal week). On the other hand, hyperthyroidism induced in rats (daily injections of T3) from the first postnatal day accelerated the growth of microglial branches and increased the density of microglial cells above normal levels [117]. Also, it has recently been shown that T3 induces microglial migration and phagocytosis, both in vitro and in vivo, via genomic and non-genomic mechanisms [118].

## 3. The Cross-Talk between Diet, Microglia, and the Endocannabinoid System

The relevance of lipids such as fatty acids (FAs) is widely recognized in the literature [119], mainly regarding to the development of the CNS. Yet, the mechanisms of action involved are still poorly understood due to the different amounts of bioactive lipid mediators that can be generated. Currently, the literature shows that dietary FAs are directly related to neuroinflammatory process influencing the microglial pro- and anti-inflammatory phenotypes [120]. Polyunsaturated fatty acids (PUFAs), n-3 FA and n-6 FA, are called essential fatty acids (EFAs) and are precursors of long-chain polyunsaturated fatty acids (LC-PUFAs), such as docosahexaenoic acid (DHA) and arachidonic acid (AA), respectively. As they share the same enzymatic machinery for the biosynthesis of its derivatives, the lipid composition of n-3 FA and n-6 FA present in the diet directly affects the production and tissue addition of DHA and AA, with anti- and pro-inflammatory activity, respectively [121].

A low EFA n-6/n-3 ratio is crucial for brain development, as well as for structural integrity, with the recommended overall intake ratio between n-6 and n-3 close to 4:1 [122]. However, in modern Western diets, it has been observed a drastic decline in the intake of PUFAs derived from n-3 FA, reaching a proportion of intake of n-6 FA derivatives 20–30-fold higher than n-3 FA [123]. Thus, changes in DHA and AA levels during the postnatal period in preterm infants, for example, may contribute to dysregulation of immune and inflammatory responses [124], leading to microglial malfunction on crucial physiological tasks. Accordingly, it has been demonstrated that the administration of DHA in a brain injury model alters the microglial function, reduces the M1 polarization and the release of pro-inflammatory cytokines [125].

Indeed, Velasco et al. demonstrated that chronic nutritional restriction of DHA promotes the rupture of topographical maps in the rat visual system during development, strongly suggesting a delay in axonal elimination [126]. The same study also demonstrated that a diet with reduced levels of DHA enhances local sprouting of intact axons in the superior colliculus (SC), induced by retinal damage. The data indicate, therefore, that chronic DHA restriction delays axonal elimination and the closure of the critical period in the visual system, directly impacting natural and lesion-induced neuroplasticity [126]. Also, DHA deprivation induces a phenotypic shift upon microglia with increasing expression of pro-inflammatory cytokines (our unpublished data). It has also been shown that oral supplementation with fish oil in a chronic DHA restriction model during early postnatal weeks was able to restore normal development [127].

In addition to a role in development, omega-3 fatty acids are also important during aging, which is characterized by an increase in pro-inflammatory cytokines in the brain leading to a microglial polarization to an M1-like profile [128]. Moreover, during aging, a change in the lipid composition of membranes is observed [129] with a resulting decrease in DHA content, which has been attributed, at least in part, to a change in the availability and functionality of lipid transportation proteins present in brain membranes [129,130]. In fact, n-3 PUFA supplementation in aged mice decreased pro-inflammatory cytokines and induced the recovery of microglial polarization, as well as a significant cognitive improvement over non-supplemented animals [128].

LC-PUFAs are also precursors of a large repertoire of bioactive lipid mediators. Arachidonic acid is the precursor of a wide range of mediators, including the two main endocannabinoids (eCBs) of the CNS, anandamide (AEA) and 2-arachidonylglycerol (2-AG). In contrast, DHA and eicosapentaenoic acid (EPA) are precursors of eCB docosahexanoyl ethanolamide (DHEA) and eicosapentaenoyl ethanolamide (EPEA), respectively [131]. The eCB system has been shown to play an important role in neuroprotective and pro-neurogenic processes, such as the attenuation of neuroinflammation, regulation of pro-inflammatory cytokine release, and increased synaptic plasticity and neurogenesis [132]. Since eCBs are lipid signaling agents produced from LC-PUFAs, strong evidence suggests that diet may lead to a change in the eCB system neuroinflammatory signaling [133,134]. Because of their fundamental nature, AA, DHA, their respective mediators and the eCB system have a large spectrum of effects on the CNS, and recent evidence strongly indicates a complex interaction among them. The levels of AA bound to phospholipids determine the levels of 2-AG and AEA that, in addition to their own biological activities, act as AA reservoirs for the subsequent production of eicosanoids, molecules that are strongly related to pro-inflammatory responses [135]. On the other hand, in addition to DHA being an important source of docosanoids known to have anti-inflammatory and pro-restorative properties [136], it is also a precursor to DHEA, which seems to be involved with the stimulation of synaptogenesis and neurite growth, 10–100 times more efficient than DHA [137].

In the CNS, eCBs are produced by neurons and glial cells, and appear to play a key role in synaptic plasticity and neuroimmune networks [138]. Recent studies have shown that brain levels of LC-PUFAs respond to diet and the ratio of n-6 FA over n-3 FA intake [133]. Alvheim et al. showed, in animal models, that diets rich in n-6 FA elevate the levels of 2-AG and AEA [139]. On the other hand, another study has shown, in mice, that a long-term dietary deficiency of n-3 FA-derived LC-PUFAs was able to abolish eCB-mediated neuronal functions in a variety of brain regions, showing that the eCB system can be regulated by the lipid composition of dietary PUFAs [140]. A recent study has also demonstrated that a two-week administration of a DHA-enriched diet is able to increase DHA and EPA concentrations, and also increase levels of the eCB DHEA and 2-eicosapentaenoylglycerol (2-EPG), and to decrease AEA levels in the brain and plasma of mice [141].

Microglial cell branches, which are in close contact with synapses and blood vessels, differentially express both CB1 and CB2 receptors. Non-activated microglia express low amounts of CB2, but levels of this expression increase strongly in neuroinflammation processes associated with brain pathologies [142]. Indeed, microglia produce approximately 20 times more eCB than astrocytes and neurons in vitro [143], so it is suggested that these cells may constitute the main cellular source of eCB under neuroinflammatory conditions [144].

The increase in microglial CB2 expression has been extensively related to several neuroprotective responses [145], such as the reduction of pro-inflammatory cytokine release [146] and modulation of migration and infiltration in inflamed brain areas or in the process of degeneration [147]. These and other actions place eCBs as promising therapeutic tools to avoid the harmful effects of inflammation, possibly through microglial modulation, generating a repairing environment in neurodegenerative conditions [148]. In addition, DHA has been linked to beneficial effects in the prevention and treatment of a wide variety of inflammatory diseases [149]. Studies with the nutritional restriction of LC-PUFAs derived from n-3 FA have shown that this dietary deficiency in the developing brain leads to a CNS pro-inflammatory state with the increase of pro-inflammatory cytokines and changes in the microglial phenotype [150].

In turn, DHA administration has the ability to prevent microglial activation towards a pro-inflammatory profile [151], demonstrating the anti-inflammatory role of this LC-PUFA, since it induces a branched and inactive microglial phenotype [152].

In summary, the n-6/n-3 FA balance in the diet seems to be essential for the correct course of CNS development since the PUFAs establish a cross-talk between the endocannabinoid system and microglia. Interestingly, both microglia and the endocannabinoid system respond to the levels of PUFAs, and low concentrations of n-3 FA in the diet induces a neuroinflammatory phenotype which seems to alter CNS development. A greater availability of n-3 FA in the diet, in turn, can alter the cannabinoid machinery, favoring the increase of the synthesis of specific eCB and the increase of cannabinoid receptor expression, mainly CB2. Signaling pathways associated with CB2 receptors in microglia, for example, converging to the acquisition of an alternative or reparative phenotype, may underlie the immunomodulatory and neuroprotective effects of eCBs on the control and restoration of CNS homeostasis.

## 4. Conclusions

**Figure 3:**
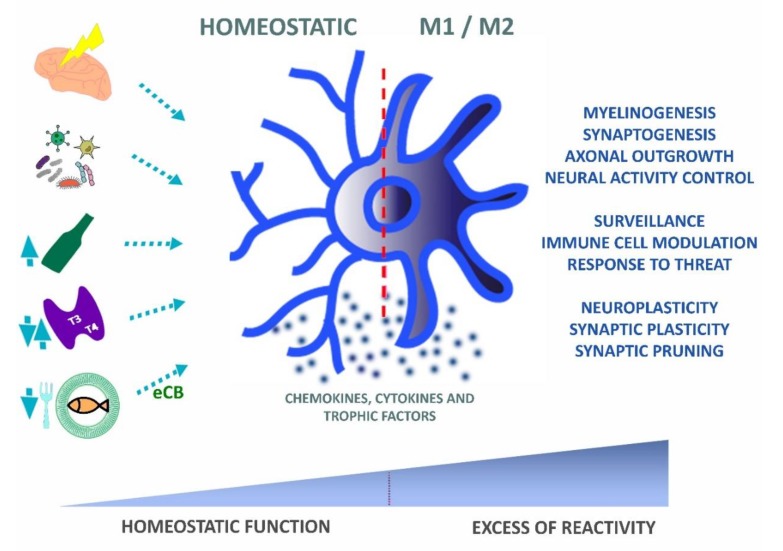
Microglial functional balance is affected by a diversity of environmental factors. Under physiological conditions, microglia interact with the microenvironment through multiple factors (chemokines, cytokines, or/and trophic factors) which modulate its functions in health and disease. As it receives multiple signals from the environment (e.g., brain trauma or injury, infection, alcohol, hormonal imbalance, or omega-3 fatty acid (FA) dietary restrictions), these cells undergo dynamic phenotypic modifications, which convert the homeostatic microglia into reactive microglia. Endocannabinoids derived from the diet act as anti-inflammatory signaling molecules that may restore microglial homeostatic functions. The phenotypic shift towards pro- and anti-inflammatory phenotypes can result in a healing process, whereas an excess of pro- over anti-inflammatory activation can result in pathological outcomes. Normal synaptic pruning and developmental circuitry remodeling depends on homeostatic microglia and, thus, an excess activation through environmental stressors may result in a loss of microglial physiological functions with possible implications on the emergence of pathological conditions such as autism and schizophrenia.

In the present review, we discussed data that describe how inflammatory responses affect brain development and plasticity. Neuroinflammation, from a wide range of environmental signals, change the behavior of microglia, affecting their physiological role during development by altering cytokine levels and the cross-talk between microglia and leukocyte populations, including T cell lymphocytes. Therefore, although microglia reactivity is necessary for healing processes after brain injury, it may also worsen the outcome of neural regeneration and induce abnormal development in conditions related to systemic maternal infection, undernutrition, hormonal imbalance, and inflammatory conditions induced by the abuse of drugs such as alcohol. An abnormal microglia function, away from a physiological set point, will directly impact the nervous system, influencing critical steps of development, such as neurogenesis, apoptosis, myelination, and the selective elimination of developing synapses (Figure 3). Therefore, developmental or neuropsychiatric disorders can be the result of those abnormal neuroimmune interactions that ultimately impact the formation of highly sensitive use-dependent neural circuitries.
